# Association between ABO blood groups and major disease categories in a retrospective cohort of 1.7 million blood donors

**DOI:** 10.7189/jogh.16.04309

**Published:** 2026-11-13

**Authors:** Liqin Wang, Shu Su, Lei Zhang, Ting Ma, Xinxin Xie, Aowei Song, Jiangcun Yang, Yang Sun

**Affiliations:** 1 Department of Transfusion Medicine, Shaanxi Provincial People’s Hospital, Xi’an, China; 2 Clinical Research Management Office, The Second Affiliated Hospital of ChongQing Medical University, Chong Qing, China; 3 China-Australia Joint Research Center for Infectious Diseases, School of Public Health, Xi’an Jiaotong University Health Science Center, Xi’an, China

**Keywords:** ABO blood groups, blood donors, cohort study, fatal diseases

## Abstract

**Background:**

ABO blood groups have a certain correlation with the incidence of many diseases. We aimed to explore the association between ABO blood groups and the top 25 fatal diseases in China.

**Methods:**

We constructed a retrospective cohort of 1,704,333 blood donors to investigate the association between ABO blood groups and the top 25 fatal diseases among Chinese individuals. We summarised differences in disease patterns across blood groups using multivariate Cox proportional hazards regression models.

**Results:**

Among the enrolled participants, type A (HR = 1.07; 95% CI = 1.03–1.09, *P* = 0.004) and type B (HR = 1.06; 95% CI = 1.02–1.09, *P* = 0.045) donors were more likely to develop ischaemic heart disease (I20–25) than type O donors. Compared with type O, type A (HR = 1.10; 95% CI = 1.06–1.13, *P* < 0.00067) and B (HR = 1.12; 95% CI = 1.08–1.14, *P* < 0.00067) donors were found to have more likely to develop diabetes mellitus (E10–14) (significant after Bonferroni correction). Type AB (HR = 1.84; 95% CI = 1.40–2.13, *P* = 0.05) and B (HR = 1.44; 95% CI = 1.19–1.71, *P* = 0.036) were more likely to develop colorectal cancer (C18–C20).Type A (HR = 0.93; 95% CI = 0.90–0.96, *P* = 0.025) and type B (HR = 0.93; 95% CI = 0.88–0.96, *P* = 0.020) were less likely to develop influenza, pneumonia, and acute lower respiratory tract infections (J09–22). Mean hospital stay differed significantly among blood groups for C18–20 (*P* = 0.045) and I20–25 (*P* = 0.008).

**Conclusions:**

We found a significant correlation between ABO blood groups and the top 25 fatal diseases in China, including ischaemic heart disease, diabetes, and colorectal cancer among blood donors. The associations should be considered exploratory and require independent validation for the general population.

**Registration:**

China National Medical Research Register: MR-61-21-011750; Chinese Clinical Trial Registry: ChiCTR2200055983

The ABO blood group system has been investigated for over a century, with various studies exploring potential links to disease susceptibility [1]. As early as 1962, a correlation between ABO blood group and ischaemic heart disease deaths was identified [2], and subsequent studies have further examined the association between ABO blood group and various diseases. For instance, individuals with blood group O have a higher risk of bleeding events but a lower risk of thromboembolic events compared to those with non-O blood groups [3–5]. These differences in disease susceptibility are believed to be attributed to varying levels of factor VIII and von Willebrand factor associated with different ABO blood groups [3,6–9].

ABO blood group antigens are terminal sugar residues located at the surface of red blood cells and various human cell and tissue surfaces, including platelets, vascular endothelium, sensory neurons, and epithelial cells [10,11]. These antigens play a critical role in transfusion medicine and are closely related to susceptibility to several diseases [1,3,12].

Over the past 30 years, China's national health plan has consistently aimed to increase life expectancy by exploring preventive strategies to reduce mortality. One promising area of research is the prevention and control of diseases that are linked to blood group susceptibility. A recent study by Dahlén *et al.* [13] analysed the medical records of 5.1 million individuals in the Swedish National Blood Donation and Transfusion Database [4,9], identifying diseases associated with ABO blood group. Despite the availability of such data, a comprehensive and well-documented spectrum of diseases linked to ABO blood group susceptibility in the Chinese population is still lacking. Therefore, we aimed to systematically investigate the association between ABO blood groups and certain diseases to provide well-documented data on blood group susceptibility and inform national health strategies in China.

Based on a report of the 25 diseases with the highest death rates in China [14], we aimed to explore the potential association between ABO blood group and these 25 diseases that exhibit the highest mortality rates among Chinese residents. To do so, we extracted electronic health records (EHR) from Shaanxi residents from January 2012 to December 2018 and classified the 25 disease codes (ICD-10). We then examined the association between these diseases and blood type in China.

## METHODS

### Data source

We used the Shaanxi Blood Donation Database, a comprehensive registration system for blood donation and transfusion in Shaanxi province, Northwest China, covering all ten prefectural districts in the region. The database includes blood information from 3.4 million voluntary blood donors, whose records were entered into the system between 1998 and 2018. The database is designed to be highly standardised, with each prefecture-level blood station adopting a consistent approach to blood collection procedures and information management. Provincial-level system administrators collect and export data from individual prefecture-level blood banks, which is then integrated into large data sets. The system has recently been updated and now includes information on 1.7 million donors (**Figure 1**). Unique individual national registration numbers assigned to all residents in the database allow for complete follow-up of all study participants by linking to the national population, death, and migration register. The Shaanxi Blood donor database has also been integrated with the Shaanxi Resident EHR [15,16], a nationwide digital health initiative implemented by the Chinese government in 2009 and widely recorded in Shaanxi since 2012.

**Figure 1:**
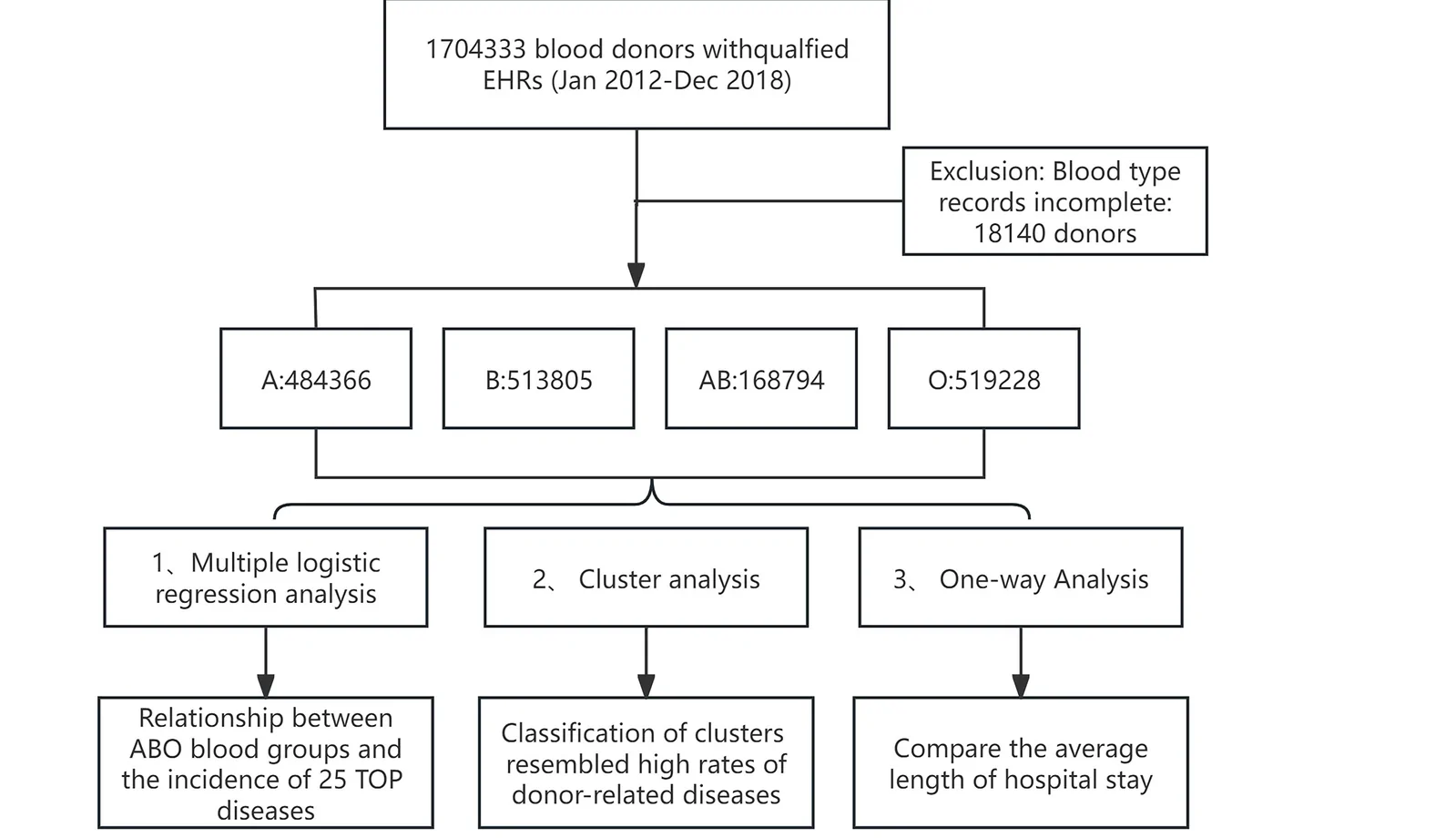
Retrospective cohort construction flowchart. The inclusion criteria for cohort participants: 1) blood donors are recorded in both the Shanxi Blood Donor’s Database and Shaanxi residents' electronic health records; 2) blood donors have a unique identification number to enable them to be linked across datasets. EHR – electronic health record.

### Selection of diseases

According to a 2017 report, the top 25 diseases associated with high mortality rates in China include: stroke (I60–67; I69), ischaemic heart disease (I20–25), tracheal, bronchial, and lung cancer (C33–34), chronic obstructive pulmonary disease (J44), Malignant neoplasm of liver and intrahepatic bile ducts (C22), road injuries (V01–99), malignant neoplasm of stomach (C16), Alzheimer disease (G30), neonatal related diseases (P00–96), hypertensive heart disease(I11, I13), oesophageal malignant tumours (C15), lower respiratory infections(J09–22), intentional self-harm (X60–84), cirrhosis, and other chronic liver diseases (K71–77), colorectal cancer (C18–20), chronic glomerulonephritis syndrome, chronic tubulointerstitial nephritis, chronic renal failure and uraemia (N03, N11, N18, N19), congenital malformations, deformations and chromosomal abnormalities (Q00–99), falling (W00–19), drowning (W65–74), diabetes mellitus (E10–14), malignant neoplasm of breast (C50), lymphatic, hematopoietic and related tissue malignant tumours (C91–96), meninges, malignant tumours of the brain, spinal cord, cranial nerves, and other parts of the central nervous system (C70–72), malignant neoplasm of pancreas (C25), and other malignant tumours (C00–90).

Other high-mortality, low-morbidity malignant tumours were classified into a separate group, including malignant neoplasms of the lips, mouth and pharynx, digestive organs, respiratory and chest organs, bone and articular cartilage, melanoma of the skin and other malignant, mesenchymal and soft tissue malignant tumours, male and female genital organs malignant tumours, urinary system malignant tumours, eye, brain, and other parts of the central nervous system (C00–90).

### Blood group test

We stratified participants by ABO blood group into four categories: A, B, AB, and O. To ensure eligibility for blood donation, donors underwent two tests to determine their ABO blood group. The first test was a rapid test performed before blood donation, and the second was a confirmatory agglutination test after each donation to ensure compatibility with the blood recipients' blood groups. Trained personnel carried out all tests at the blood stations. The blood group detection and information management system used in all the prefecture-level city blood stations was standardised.

### Inclusion and exclusion criteria

We included individuals aged 18–70 years with documented ABO blood groups and a hospitalisation record for any of the top 25 diseases during the study period. We also included participants with multiple hospitalisations for the same disease only for their first hospitalisation record. We excluded records with duration <1 day or >180 days (extreme outliers).

### Data analysis

In this blood donor cohort, we collected comprehensive health information, including hospitalisation records, between 2012 and 2018. We performed descriptive and inferential statistical analyses to investigate the relationship between ABO blood groups and the various diseases among blood donors. Numerical variables were summarised using mean (x̄) and standard deviation (SD), while categorical variables were described using frequency and percentage. We used the χ^2^ test to determine if there were any significant sex differences in basic demographic characteristics. Additionally, we used one-way analysis of variance to compare the average length of hospital stay among patients with different ABO blood groups. We used multivariate Cox proportional hazards regression models to evaluate associations between ABO blood groups and the risk of various diseases, with blood type O as the reference group. We calculated hazard ratios (HRs) and their corresponding 95% confidence intervals (CI) as the primary effect estimates, adjusted for age (18–35, 36–54, ≥55 years), sex, donation centre, donation frequency, residence (urban/rural), and occupation. When the HR is >1, it indicates an adverse prognostic factor (increased risk); when the HR is <1, it suggests a protective factor (reduced risk). Given 75 comparisons (25 diseases ×3 blood type contrasts), Bonferroni correction set the significance threshold at *P* < 0.00067. Associations with *P* < 0.05 but ≥0.00067 are considered exploratory and require independent validation. We grouped different conditions within different ABO blood groups by x̄ length of stay using a similarity scale, with Euclidean distance as the measure of similarity. Euclidean distance represents the distance between diseases and can be measured in two- or three-dimensional Euclidean space, where a smaller value indicates a closer relationship between two properties. We divided data sets into clusters based on the nearest Euclidean distance value. We used a Bonferroni correction for 25 tests (the number of health outcomes for each blood group) to adjust for multiple testing, with *P* < 0.002 considered statistically significant. We performed all other statistical tests with a significance level of 0.05.

We analysed the data using *R*, version 4.1.2 (R Core Team, Vienna, Austria). Finally, all donors provided informed consent to donate blood.

## RESULTS

### Demographic characteristics

We included 1,018,951 men and 685,382 women from the Shaanxi provincial EHR system (**Table 1**). On average, men (x̄ = 29.52, SD = 14.00) were older than women (x̄ = 30.20, SD = 11.99) within the study population (*P* < 0.001). More than 64% of both men and women were in the 18–35 age range. Almost all women (98.1%) and men (98.69%) were of the Han ethnic group. About half of women (48.94%) and slightly over half of men (55.11%) were married, while 40.35% of women and 34.75% of men had college degrees or higher. Students accounted for 22.05% of women and 15.15% of men, mostly in Xi'an. Sex distribution differed significantly among the four blood groups (*P* < 0.001). The smallest proportion of participants had an AB blood type, approximately 10%, while the other three blood types each accounted for about 30%. The average number of blood donations was higher for men (x̄ = 1.8, SD = 0.8) compared to women (x̄ = 1.6, SD = 0.9) (*P* < 0.001).

**Table 1:** Demographic characteristics of blood donors in Shaanxi province*

	Men	Women	*P*-value
**Number of participants**	1,018,951 (59.79)	685,382 (40.21)	
**Age in years**			<0.001
18–35	384,423 (64.68)	596,701 (64.93)	
36–54	200,323 (33.70)	310,725 (33.81)	
≥55	9,601 (1.62)	11,503 (1.25)	
**Average donation counts, x̄ (SD)**	1.8 (0.8)	1.6 (0.9)	<0.001
**Blood types**			<0.001
A	194,222 (28.53)	290,144 (28.86)	
B	207,682 (30.51)	306,123 (30.45)	
AB	68,200 (10.02)	100,594 (10.00)	
O	210,614 (30.94)	308,614 (30.69)	
**Ethnicity**			<0.001
Han	672,368 (98.10)	1,005,634 (98.69)	
Non-Han	13,014 (1.90)	13,317 (1.31)	
**Education**			<0.001
Junior high and below	183,266 (27.77)	294,834 (29.94)	
Senior high	210,454 (31.89)	347,643 (35.31)	
University and above	266,285 (40.35)	342,142 (34.75)	
**Marital status**			<0.001
Single	302,093 (51.06)	400,623 (44.89)	
Married	289,516 (48.94)	491,742 (55.11)	
**Occupation**			<0.001
Student	145,316 (22.05)	148,901 (15.15)	
Worker	176,390 (26.76)	300,760 (30.60)	
Peasant	80,426 (12.20)	148,763 (15.14)	
Self-employed	256,950 (38.99)	384,478 (39.12)	
**Region**			<0.001
Ankang	45,510 (6.64)	65,232 (6.40)	
Baoji	59,843 (8.73)	99,792 (9.79)	
Hanzhong	69,024 (10.07)	99,570 (9.77)	
Shangluo	23,754 (3.47)	33,569 (3.29)	
Tongchuan	14,909 (2.18)	19,438 (1.91)	
Weinan	55,174 (8.05)	103,763 (10.18)	
Xian	303,104 (44.22)	419,083 (41.13)	
Xianyang	89,024 (12.99)	132,457 (13.00)	
Yanan	24,949 (3.64)	45,854 (4.50)	
Yulin	91 (0.01)	193 (0.02)	

SD – standard deviation, x̄ – mean

*Values are presented as n (%) unless specified otherwise.

### Disease progression between ABO blood groups

We analysed the relationship between ABO blood groups and the risk of disease onset using multivariate Cox proportional hazards regression (**Table 2**). Compared with type O, type A (HR = 1.10; 95% CI = 1.06–1.13, *P* < 0.00067) and B (HR = 1.12; 95% CI = 1.08–1.14, *P* < 0.00067) donors were found to be more likely to develop diabetes mellitus (E10–14) after Bonferroni correction. Additionally, type A (HR = 1.07; 95% CI = 1.03–1.09, *P* = 0.004) and B (HR = 1.06; 95% CI = 1.02–1.09, *P* = 0.045) donors had a higher likelihood of experiencing ischaemic heart diseases (I20–25) when compared with type O donors prior to Bonferroni correction. Type AB (HR = 1.84; 95% CI = 1.40–2.13, *P* = 0.05) and B (HR = 1.44; 95% CI = 1.19–1.71, *P* = 0.036) donors had an increased likelihood of developing colorectal cancer (C18–20). Conversely, individuals with A (HR = 0.93; 95% CI = 0.90–0.96, *P* = 0.025) and B (HR = 0.93; 95% CI = 0.88–0.96, *P* = 0.020) blood groups were less likely to develop influenza, pneumonia, and acute lower respiratory tract infections (J09–22).

**Table 2:** Multivariate Cox proportional hazards analysis of ABO blood group-related disease risk

	Type A		Type B		Type AB	
**Disease name (code)**	**HR (95% CI)**	***P*-value**	**HR (95% CI)**	***P*-value**	**HR (95% CI)**	***P*-value**
Stroke (I60–67; I69)	1.01 (0.95–1.07)	0.843	0.97 (0.94–1.02)	0.615	0.96 (0.94–1.03)	0.311
Ischaemic heart diseases (I20–25)	1.07 (1.03–1.09)	0.004*	1.06 (1.02–1.09)	0.045*	1.11 (1.05–1.14)	0.195
Tracheal, bronchial, and lung cancer (C33, C34)	1.04 (0.93–1.23)	0.643	1.07 (0.92–1.25)	0.651	0.91 (0.72–1.16)	0.701
Chronic obstructive pulmonary disease (J44)	0.94 (0.82–1.07)	0.625	1.11 (0.98–1.27)	0.396	1.08 (0.90–1.29)	0.624
Malignant neoplasm of liver and intrahepatic bile ducts (C22)	1.43 (0.91–2.25)	0.182	1.20 (0.90–1.59)	0.521	0.90 (0.61–1.45)	0.834
Malignant neoplasm of stomach (C16)	1.19 (0.97–1.33)	0.340	0.92 (0.75–1.09)	0.709	0.75 (0.53–1.11)	0.513
Alzheimer disease (G30)	NA	NA	1.93 (0.67–5.48)	0.526	NA	NA
Neonatal-related diseases (P00–96)	0.92 (0.83–1.08)	0.401	1.03 (0.94–1.09)	0.437	1.03 (0.87–1.14)	0.825
Hypertensive heart disease (I11, I13)	0.97 (0.76–1.25)	0.901	0.77 (0.58–1.01)	0.305	0.68 (0.45–1.03)	0.292
Oesophageal malignancies (C15)	0.87 (0.57–1.27)	0.645	1.25 (0.89–1.43)	0.413	1.43 (0.96–1.95)	0.153
Influenza, pneumonia, acute lower respiratory tract infections (J09–22)	0.93 (0.90–0.96)	0.025*	0.93 (0.88–0.96)	0.020*	0.95 (0.93–1.01)	0.442
Intentional self-harm (X60–84)	1.14 (0.83–1.53)	0.685	1.29 (0.94–1.75)	0.451	0.70 (0.47–1.18)	0.480
Cirrhosis and other chronic liver diseases (K71–77)	0.95 (0.91–1.08)	0.651	0.96 (0.89–1.06)	0.927	0.98 (0.91–1.08)	0.878
Colorectal cancer (C18–20)	1.28 (0.97–1.49)	0.093	1.44 (1.19–1.71)	0.036*	1.84 (1.40–2.13)	0.005*
Chronic glomerulonephritis syndrome, chronic tubulointerstitial nephritis, chronic renal failure and uremia (N03, N11, N18, N19)	0.89 (0.76–1.04)	0.059	0.92 (0.83–1.00)	0.404	0.85 (0.71–0.99)	0.060
Congenital malformations, deformations and chromosomal abnormalities (Q00–99)	1.03 (0.97–1.09)	0.451	0.98 (0.91–1.05)	0.735	0.87 (0.80–1.02)	0.272
Diabetes mellitus (E10–14)	1.10 (1.06–1.13)	<0.00067†	1.12 (1.08–1.14)	<0.00067†	1.06 (1.01–1.10)	0.067
Malignant neoplasm of breast (C50)	0.86 (0.75–0.98)	0.103	0.89 (0.80–1.08)	0.121	1.09 (0.95–1.29)	0.352
Lymphatic, hematopoietic and related tissue malignancies (C91–96)	0.76 (0.64–1.01)	0.083	0.88 (0.67–1.05)	0.499	0.74 (0.56–1.01)	0.315
Meninges, malignant tumours of the brain, spinal cord, cranial nerves, and other parts of the central nervous system (C70–72)	1.05 (0.80–1.37)	0.921	1.27 (0.97–1.77)	0.316	1.10 (0.77–1.70)	0.746
Malignant neoplasm of pancreas (C25)	0.57 (0.36–1.01)	0.299	1.00 (0.67–1.53)	0.950	1.46 (0.88–2.35)	0.392
Malignant neoplasms (C00–90)	1.09 (1.00–1.17)	0.162	1.01 (0.95–1.11)	0.788	0.95 (0.87–1.05)	0.762

CI – confidence interval, HR – hazard ratio, NA – not applicable

*Significant (*P* < 0.05) before Bonferroni correction.

†Significant (*P* < 0.00067) after Bonferroni correction.

### Duration of hospitalisation by ABO blood groups

The x̄ length of hospital stay was the shortest for neonatal-related illnesses (P00–96) and intentional self-harm (X60–84) (**Figure 2**; **Table 3**). The average length of hospital stay for oesophageal malignant tumours (C15) (*P* = 0.383) and lymphatic, hematopoietic and related tissue malignant tumours (C91–96) (*P* = 0.703) differed by blood group, but the differences were not statistically significant. The x̄ length of stay for colorectal cancer (C18–20) (*P* = 0.045) and ischaemic heart disease (I20–25) (*P* = 0.008) differed significantly among blood groups. There were not enough patients to analyse Alzheimer disease (G30).

**Figure 2:**
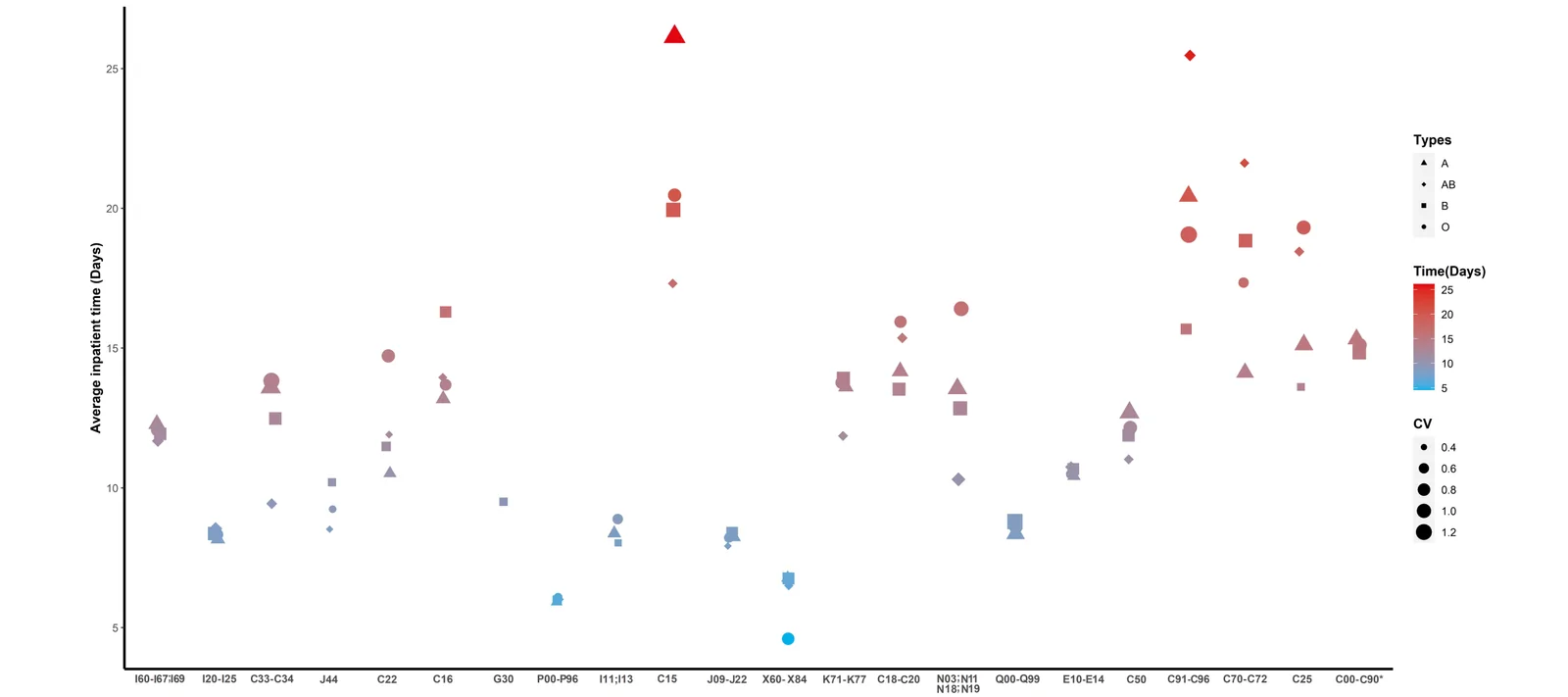
Average hospitalisation days of different ABO blood groups in different diseases. CV – coefficient of variation.

**Table 3:** Average length of hospital stays for different diseases by blood type, x̄ (SD)

Disease name (code)	Type A	Type AB	Type B	Type O	*P*-value
Stroke (I60–67, I69)	11.67 (7.87)	11.39 (9.44)	11.26 (8.51)	11.62 (8.79)	0.345
Ischaemic heart diseases (I20–25)	8.06 (5.39)	8.14 (5.57)	8.29 (4.36)	8.28 (4.10)	0.008*
Tracheal, bronchial, and lung cancer (C33, C34)	15.27 (9.20)	10.61 (5.38)	13.82 (8.04)	14.79 (10.53)	0.357
Chronic obstructive pulmonary disease (J44)	9.13 (3.33)	8.09 (2.67)	9.49 (3.93)	9.01 (4.42)	0.423
Malignant neoplasm of liver and intrahepatic bile ducts (C22)	11.94 (6.59)	11.10 (6.27)	11.03 (5.38)	11.29 (5.26)	0.954
Malignant neoplasm of stomach (C16)	14.54 (8.59)	18.25 (7.62)	18.31 (14.07)	16.23 (8.70)	0.118
Alzheimer disease (G30)	8.00 (2.67)		9.50 (4.50)	2.00 (1.00)	
Neonatal-related diseases (P00-P96)	5.76 (3.98)	6.01 (3.06)	5.92 (3.06)	6.09 (3.55)	0.538
Hypertensive heart disease (I11, I13)	8.39 (4.43)	8.78 (2.07)	7.26 (3.04)	8.69 (4.32)	0.666
Oesophageal malignancies (C15)	26.19 (20.99)	16.89 (12.35)	23.53 (19.41)	12.67 (8.08)	0.383
Influenza, pneumonia, acute lower respiratory tract infections (J09–22)	8.31 (4.86)	7.87 (4.31)	8.21 (4.84)	8.20 (5.07)	0.173
Intentional self-harm (X60–84)	6.18 (3.56)	6.50 (4.04)	6.44 (3.26)	4.20 (2.26)	0.477
Cirrhosis, and other chronic liver diseases (K71–77)	14.15 (9.26)	10.82 (6.28)	13.69 (8.99)	13.02 (8.31)	0.075
Colorectal cancer (C18–20)	13.72 (7.80)	19.19 (10.42)	17.18 (13.17)	17.47 (11.03)	0.045*
Chronic glomerulonephritis syndrome, chronic tubulointerstitial nephritis, chronic renal failure and uremia (N03, N11, N18, N19)	10.75 (7.63)	11.06 (6.47)	11.22 (7.85)	11.67 (9.14)	0.560
Congenital malformations, deformations and chromosomal abnormalities (Q00–99)	7.80 (5.85)	7.63 (4.73)	9.08 (4.80)	8.87 (6.59)	0.366
Diabetes mellitus (E10–14)	10.53 (4.92)	10.68 (4.90)	10.66 (4.53)	10.45 (4.90)	0.941
Malignant neoplasm of breast (C50)	14.94 (10.74)	12.76 (6.85)	14.66 (9.44)	14.22 (6.48)	0.820
Lymphatic, hematopoietic and related tissue malignancies (C91–96)	23.29 (18.33)	29.33 (20.71)	17.11 (12.58)	23.62 (15.92)	0.703
Meninges, malignant tumours of the brain, spinal cord, cranial nerves, and other parts of the central nervous system (C70–72)	15.72 (10.16)	21.18 (13.74)	25.48 (21.51)	15.83 (8.06)	0.208
Malignant neoplasm of pancreas (C25)	15.67 (9.14)	22.00 (15.99)	14.58 (7.27)	14.46 (11.82)	0.530
Malignant neoplasms (C00–90)	15.61 (12.36)	16.43 (10.71)	15.79 (11.50)	15.65 (9.39)	0.871

SD – standard deviation, x̄ – mean

*Significant (*P <* 0.05) before Bonferroni correction.

We investigated disease cluster classification based on Euclidean distance and number of days in the hospital for donors of different ABO blood groups (**Figure 3**). Two disease clusters were identified: Cluster 1 included tracheal, bronchial, and lung cancer (C33–34), congenital malformations, deformations and chromosomal abnormalities (Q00–99), lymphatic, hematopoietic, and related tissue malignant tumours (C91–96), chronic kidney diseases, oesophageal malignant tumours (C15), meninges, malignant tumours of the brain, spinal cord, cranial nerves, and other parts of the central nervous system (C70–72), chronic obstructive pulmonary diseases (J44), malignant tumours of the liver and intrahepatic bile duct (C22), and neonatal related diseases (P00–96). Participants with type AB were found to have a higher risk of developing diseases in Cluster 1 compared to type O. In Cluster 2, participants with blood type AB had a lower risk than those with blood type O.

**Figure 3:**
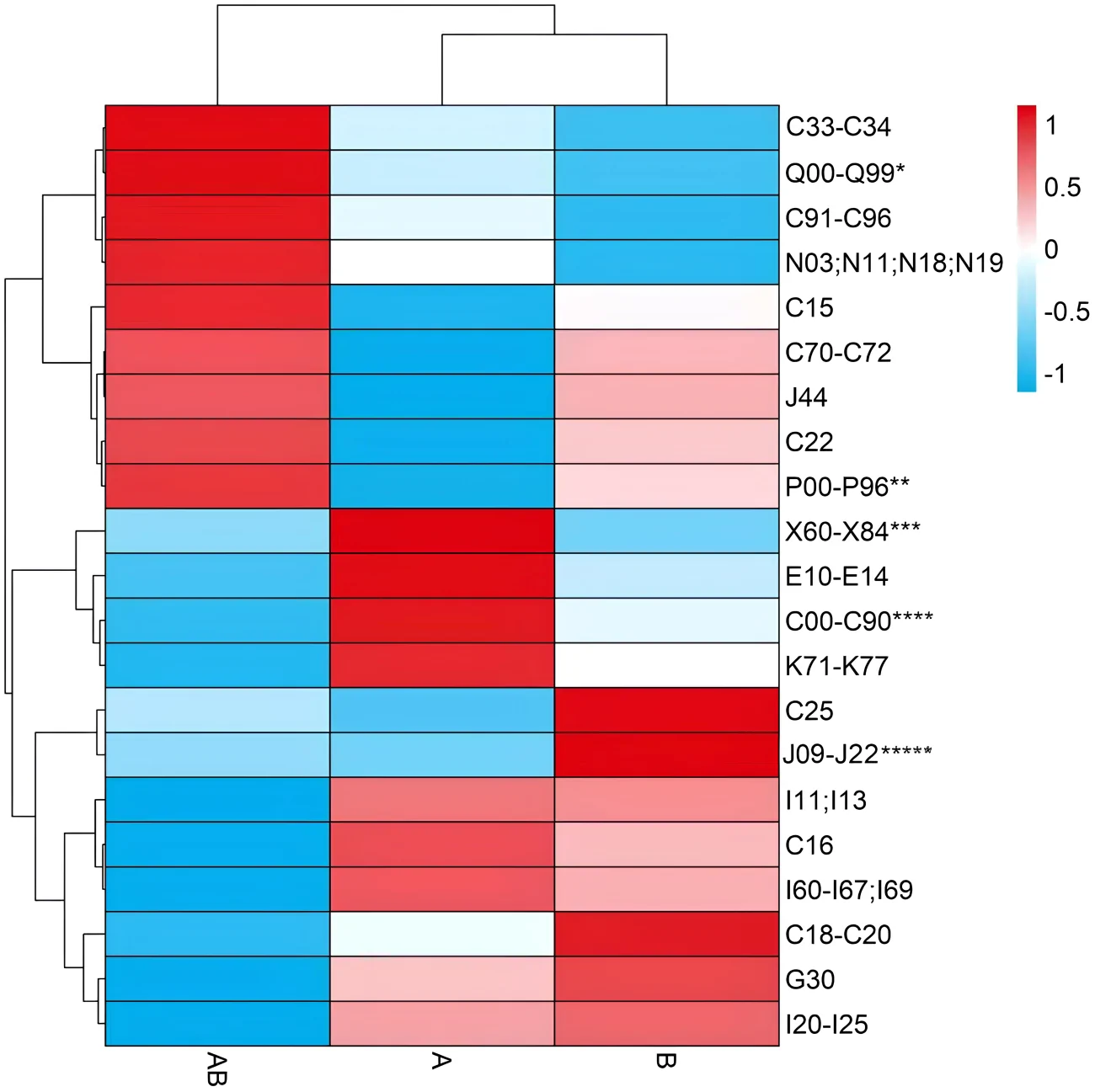
Heatmap of clustering according to average hospitalisation days.

## DISCUSSION

We found that ABO blood groups are linked to certain diseases. Individuals with blood type A and B were more prone to developing ischaemic heart disease and diabetes compared to those with blood type O. Individuals with blood type AB and B had a higher risk of developing colorectal malignant tumours. We also examined the duration of hospital stays for different diseases among ABO blood groups. Overall, patients with blood type A had the shortest hospital stay for ischaemic heart disease, while patients with blood type B had the longest. In contrast, patients with blood type AB had the longest hospital stay for colorectal malignant tumours, while patients with blood type A had the shortest. Individuals with blood type A and B have a lower incidence of influenza, pneumonia, and acute lower respiratory tract infections.

The ABO blood groups' risk of 25 kinds of diseases among ABO blood groups (O and non-O blood groups) was discussed using multivariate Cox proportional hazards regression. Based on the exploration of risk factors, we found differences in the incidence rate of malignant tumours among different ABO blood groups. We also found that, compared with blood type O, type AB is more prone to respiratory and liver diseases, and type A is more prone to diabetes and cerebrovascular diseases.

Ischaemic heart disease is the second most common cause of death in China, and the potential link between blood type and the severity of the disease or mortality has been extensively studied. We discovered notable differences in the risk of ischaemic heart disease among individuals with different ABO blood groups. Except for blood type A, blood type B is also the most susceptible to ischaemic heart disease. In terms of hospital stay duration, patients with type A blood had the shortest length of stay for ischaemic heart disease, while those with type B had the longest. Although the relationship between ABO blood groups and ischaemic heart disease has been widely studied [3,5,17–19], no definitive conclusion has been reached. It has been suggested that non-O individuals have a higher incidence of cardiovascular events than those with type O blood, which our findings largely support. Although ischaemic heart disease is a complex condition with multiple factors involved, our results could still be useful in identifying individuals who may be at greater risk. Compared with type O, the susceptibility of type B to ischaemic heart disease, except for type A, deserves attention and research.

The number of people with diabetes in China has surpassed 140 million, making it the highest in the world. The situation regarding disease prevention and control is extremely concerning. We found that individuals with blood type A and B have a higher risk of developing diabetes than those with blood type O. This finding was consistent with our observations on ischaemic heart disease. This could be a coincidence, or there could be a relationship between the two conditions. Diabetes may have been screened during the diagnosis of ischaemic heart disease. Dahlen *et al*. [12] previously described this non-causal mechanistic drive, but it requires further exploration. Several epidemiological and genetic studies have investigated the association between ABO blood groups and type 2 diabetes, but the results have been inconsistent [20–23]. The mechanism depends on the levels of endothelial cell markers in different blood groups [24–26]. The controversy may be due to the retrospective design, sample size, and genetic variation among different ethnic populations. Therefore, further studies in other ethnic populations are required to validate our findings. These studies suggest that the association between ABO blood groups and diabetes risk may reflect multiple risk factors. By investigating the relationship between blood type and diabetes, we can enhance public knowledge of the disease, support early screening, and, to some extent, improve health outcomes for diabetes patients.

The link between blood groups and lower respiratory infections was highlighted by the outbreak of COVID-19 in 2019, and the link between COVID-19 and ABO blood groups has been well studied even though only three years have elapsed since the disease emerged. We found that people with blood groups A and B were less likely to develop influenza, pneumonia, and acute lower respiratory tract infections(J09–22). This result is consistent with our previous study [27] but contradicts the conclusion that blood groups A and B may be risk factors for COVID-19 [28–30]. Several studies [31,32] have not shown a relationship between blood type and disease susceptibility or with disease severity. ABO antigens were found to affect the binding of human salivary mucus glycoproteins to influenza viruses [33]. It is now well established that anti-A and anti-B antibodies act as protective factors by preventing infection of target cells through binding to the respective antigens expressed on the viral envelope or spike protein [34]. Although contrary to the results of our current study, it also suggests more complex potential associations between blood type and lower respiratory tract infection.

We found that type AB and B individuals had a higher risk of colorectal malignant tumours. In terms of length of hospital stays, patients with type AB had the longest stay, while patients with type A had the shortest. Clinical colorectal diagnosis has been in the middle and late stages, making early detection and prevention crucial; however, there has been little epidemiological exploration of the relationship between blood type and colorectal cancer. Some studies have suggested that type A has a greater risk of colorectal cancer than non-A blood types [35–38]. They suggested that cell proliferation and motility are highly correlated with the expression of the A blood type antigen [35,36]. However, other studies have not shown a relationship between the ABO blood groups and colorectal cancer [39,40]. The maturation hypothesis between blood groups and tumours is that ABO glycosyltransferase activity may influence the processing and clearance of molecules involved in tumorigenesis [41]. On the other hand, blood-group antigens may play a role in tumorigenesis, metastasis, and prognosis through their involvement in cell recognition, cell signalling, and cell adhesion [42]. We found that people with AB and B blood types have a higher risk of colorectal cancer. It is worth exploring whether B antigen expression is related to colorectal cancer development. Further research is needed to validate our findings and better understand the relationship between blood type and colorectal cancer; we also found little association between blood group and breast, stomach, urinary, brain, spinal cord, cranial nerves, lymphatic, hematopoietic, and other malignant tumours.

As one of many human genetic characteristics, blood group is not a decisive factor in determining disease susceptibility. Moreover, blood group distribution is affected by genetic inheritance, human migration, and regional variations. Some diseases may have evolved to target specific blood groups rather than blood groups being the determining factor for disease. With advances in molecular biology, genetics, clinical medicine, and related disciplines, the correlation between blood groups and disease will be increasingly elucidated to benefit humanity. While blood groups were found to be associated with certain diseases, this association is not absolute. The recent discoveries suggest biological mechanisms linking disease pathogenesis to ABO antigen expression. The evolution and distribution of ABO blood groups are linked to *Plasmodium falciparum* infection [43].

In modern society, numerous complex factors influence an individual's disease risk and severity, and blood type is just one. Instead of worrying about blood groups, adopting a healthy lifestyle is a more productive approach.

This is, to our knowledge, the largest study to date on the relationship between ABO blood groups and diseases in China, undertaken to discover new links and confirm previously known ones. The data presented here have several unique advantages. We selected a city with a permanent population of 39 million and conducted a 30-year retrospective study of 1.7 million blood donors’ records. We matched these data with Shaanxi resident EHRs to ensure reliable, generalisable results.

This study had several limitations. First, the included participants were only blood donors in good health, which may have prevented detection of uncommon or early-stage diseases. Second, we did not consider the participants' family disease history and other predisposing factors, both of which could have affected the outcomes. Third, the healthy donor effect limits universality. Our cohort mainly consists of young people (average age = 30), who are healthier than the general population. Therefore, the association with diseases that occur in the elderly (***e.g.*** Alzheimer disease and chronic obstructive pulmonary disease) should be interpreted with caution [44]. Additionally, the survey results from blood donors in Shaanxi Province may not fully represent the general population in China. Furthermore, diseases such as neonatal diseases (P00–P96), congenital malformations (Q00–99), and external injuries (such as V01–99, X60–84) in adult blood donors may reflect coding artefacts or family record associations. One possible reason is that newborns and the elderly may use their family members' medical insurance cards in extreme situations rather than reflecting true biological associations. Future studies of this type should pre-screen the rationality of disease categories and perform pre-exclusion before analysis. Finally, after disease screening, the baseline health level of the blood donor population was better than that of the general community population. Therefore, our results on the disease distribution and association analysis apply only to healthy blood donors and cannot be directly generalised to the general population.

## CONCLUSIONS

We identified associations between ABO blood group and several major fatal diseases in China, including ischaemic heart disease, diabetes, colorectal cancer, and lower respiratory tract infection. Only the association with diabetes remained significant after Bonferroni correction, while other associations are exploratory. Moreover, blood type was associated with differences in hospital length of stay for colorectal cancer and ischaemic heart disease. These findings are exploratory and require validation in community-based cohorts with stronger confounding control before clinical application

## Data Availability

**Data availability:** The datasets generated and analysed during the current study are available from the corresponding author upon reasonable request.
